# Tickling the TLR7 to cure viral hepatitis

**DOI:** 10.1186/1479-5876-12-129

**Published:** 2014-05-14

**Authors:** Emily Funk, Shyam Kottilil, Bruce Gilliam, Rohit Talwani

**Affiliations:** 1 Critical Care Medicine Department, Clinical Research Center, National Institutes of Health, Bethesda, MD 20892, USA; 2 Laboratory of Immunoregulation, National Institute of Allergy and Infectious Diseases, National Institutes of Health, Bethesda, MD 20892, USA; 3 Institute of Human Virology at the University of Maryland School of Medicine, 725 West Lombard St. N151, Baltimore, MD 21201, USA

**Keywords:** Hepatitis C, Hepatitis B, Toll-like receptors, TLR7, Hepatitis treatment

## Abstract

Chronic hepatitis B and C are the leading causes of liver disease and liver transplantation worldwide. Ability to mount an effective immune response against both HBV and HCV is associated with spontaneous clearance of both infections, while an inability to do so leads to chronicity of both infections. To mount an effective immune response, both innate and adaptive immune responses must work in tandem. Hence, developing protective immunity to hepatitis viruses is an important goal in order to reduce the global burden of these two infections and prevent development of long-term complications. In this regard, the initial interactions between the pathogen and immune system are pivotal in determining the effectiveness of immune response and subsequent elimination of pathogens. Toll-like receptors (TLRs) are important regulators of innate and adaptive immune responses to various pathogens and are often involved in initiating and augmenting effective antiviral immunity. Immune-based therapeutic strategies that specifically induce type I interferon responses are associated with functional cure for both chronic HBV and HCV infections. Precisely, TLR7 stimulation mediates an endogenous type I interferon response, which is critical in development of a broad, effective and protective immunity against hepatitis viruses. This review focuses on anti-viral strategies that involve targeting TLR7 that may lead to development of protective immunity and eradication of hepatitis B.

## Introduction

Defense against chronic viral infections is complex and involves both host innate and adaptive immune systems [1]. The major characteristic of the human immune system is the nature by which immune cells recognize self from non-self [2,3]. While the adaptive immune system features specific antigen recognition receptors, the innate immune system utilizes a unique set of pattern recognition receptors (PRRs), such as TLRs, which recognize non-specific, but conserved molecular structures on microorganisms [3-5]. However, certain viral pathogens such as hepatitis B (HBV) and C (HCV) may effectively evade host immune responses, thereby establishing chronic persistent infection [6,7] These infections can be treated with modest success using immunomodulatory therapy such as exogenous interferon [8-10]. Hence, there is a continued interest in other novel immune-based therapies for chronic viral hepatitis, which may improve treatment outcomes. Activation of specific TLRs results in an endogenous interferon response leading to the production of antiviral, proinflammatory and costimulatory cytokines, enabling crosstalk between the innate and adaptive immune systems, which culminates in the stimulation of immune effector cells integral to antiviral immunity [11,12]. In this regard, interactions between viruses and TLRs may play a major role in developing protective antiviral immunity. Therefore, exogenous activation of TLRs represents an attractive therapeutic strategy to combat chronic viral pathogens such as HBV and HCV.

## Review

### The toll-like receptor family

Members of the TLR family detect a wide range of conserved pathogen-associated molecular patterns (PAMPs). Upon recognition and binding to a PAMP, stimulation induces shared signaling pathways that culminate in expression of numerous host defense genes. In humans, there are 10 known members of the TLR family, which are membrane-bound proteins structurally characterized by the presence of a conserved intracellular Toll/interleukin (IL)-1 receptor (TIR) domain and leucine-rich repeat ectodomain [13,14]. This extracellular/endosomal leucine-rich domain plays a key role in the binding of ligands, although the molecular basis for the recognition of the PAMPs by TLRs is largely unknown [15]. The TLR subgroups are classified according to the location of the receptor and its function upon activation. TLRs expressed on the cell surface include TLR1, TLR2, TLR4, TLR5 and TLR6; these subgroups are able to detect bacterial and fungal cell wall components and some viral proteins [16]. The intracellular subgroups TLR3, TLR7, TLR8, and TLR9 are located within endosomes and bind to viral nucleic acids [16,17]. Their location within endosomes provides an environment in which host DNA should not be present, thus avoiding the possibly deleterious effects of self-recognition [18]. TLR3, TLR 7/8 and TLR9 recognize viral or synthetically derived double stranded RNA, viral single stranded RNA or bacterial unmethylated CpG DNA, respectively [17,19-21]. While both TLR7 and 8 have similar structures, are triggered by viral single stranded RNA, and propagate signaling via the same adapter protein, the myeloid differentiation primary-response protein (MyD88, see below), their activation appears to result in different host cell responses. Interestingly, recent studies show a vital role of TLR7 activation in early immune responses and throughout progression of plasmodial infections, another pathogen with a liver life cycle [22]. Studies utilizing synthetic agonists demonstrated higher type I interferon induction by TLR7 signaling and greater proinflammatory cytokine induction via TLR8 activation [23]. In this review, we will focus primarily on TLR7, as the majority of small molecule TLR agonists undergoing clinical development for treatment of viral hepatitis target TLR7 agonists to induce an endogenous interferon response.

#### TLR7 signaling: induction of antiviral cytokines

**Figure 1:**
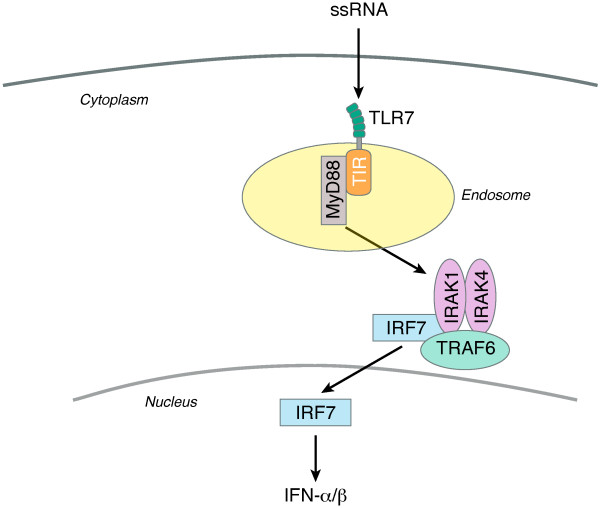
**Selective TLR7 signaling.** Natural ligands of TLR7 include ssRNA, particularly viral particles (HCV). Once an HCV virion enters the hepatocyte, it engages the TLR7 receptor resulting in the formation of a complex facilitated by the recruitment of adapter protein, MyD88 and TIR, which occurs in the endosomal compartment. This complex then triggers a series of signal transduction pathways culminating in the formation of IRAK/IRF7/TRAF6 complex. IRF7 will translocate to the nucleus and transactivate the production of type I interferons (IFN-α and β). Exclusive TLR7 agonists generate activation of type I interferons, while TLR7/8 agonists result in the generation of proinflammatory cytokines, such as IL-2, IL-8, and TNF along with interferon α/β, which may result in development of undesirable adverse events. Hence, selective TLR7 agonists are ideal in the treatment of hepatitis viruses.

TLR7 signaling is primarily associated with generation of an endogenous type I interferon response (Figure 1). Binding of TLR7 to its respective ligand causes conformational changes and dimerization of the receptors, followed by the recruitment of its specific TIR adaptor protein, MyD88. MyD88 activates a series of signal transduction molecules, including IL-1R associated kinases (IRAKs), tumor necrosis factor receptor (TNFR)-associated factor 6 (TRAF6), and transforming growth factor (TGF)-B activated kinase (TAK1), which yield activation of the transcription factors nuclear factor kappa B (NF-**κ**B) and IFN regulatory factor 7 (IRF7) (Figure 1). TLR7 signaling is biased toward IRF7 activation, which facilitates production of antiviral cytokines, including type I and type II interferons, while NF-**κ**B induces a proinflammatory effect via secretion of cytokines such as TNF-α, interleukin-6 and interleukin-12 (IL-6, IL-12) [16,18,24].

TLR7-induced production of type I and II interferons activate pathways that lead to the destruction of intracellular pathogens, including stimulation of adaptive immune effector cells [12]. This augmentation of innate and adaptive immune signaling makes TLR ligands an attractive addition to antiviral therapeutics [25]. However, immune activation can be a double-edged sword; exogenous immunomodulatory treatment must be utilized with caution in patients with cirrhosis as profibrogenic chemokine stimulation can activate hepatic stellate cells, which may exacerbate fibrosis.

#### Linking innate and adaptive immunity: TLR-controlled DC-mediated activation of T cells

**Figure 2:**
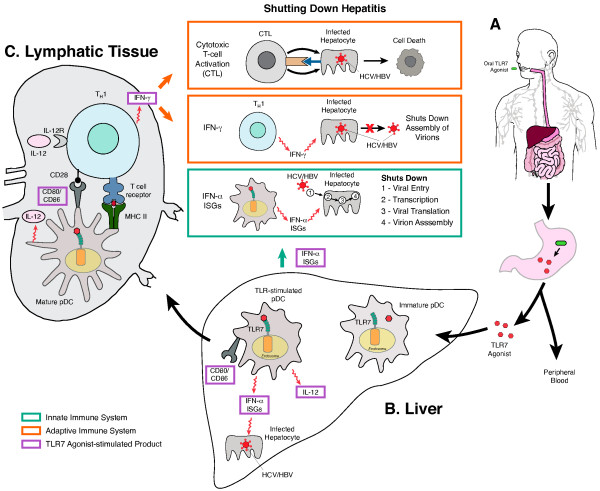
**Selective TLR 7 agonists in chronic viral hepatitis.** Selective oral TLR7 agonists are absorbed through the gastrointestinal mucosa and enter the liver through portal circulation **(A)**. In the liver, they lead to priming of immature DCs to mature DC secreting interferon-α. Interferon-α stimulation will lead to the synthesis of interferon-stimulated gene (ISG) products, which in turn facilitate suppression of hepatitis viral replication (green insert) **(B)**. Although, the exact mechanism of how interferon-α suppresses hepatitis replication is not fully characterized, multiple stages of hepatitis replication (such as viral entry, transcription, translation and assembly) have been shown to be inhibited by interferon-α and ISGs. In the lymphatics, TLR7 stimulated mature DCs secrete IL-12 and preferentially activate T cells to differentiate into T_H_1 cells **(C)**. A T_H_1 response leads to the generation of cytotoxic T cell response (red inserts). IFN-γ primed CTLs cause suppression of hepatitis viruses by either killing infected hepatocyte or by shutting down the virion assembly.

As a first line of defense, innate immune responses prime the adaptive immune system to mount an effective pathogen specific immune response. Efficient priming of adaptive immune response requires antigen presentation by the major histocompatibility complex (MHC) on antigen-presenting cells (APCs) in addition to the simultaneous induction and presentation of accessory signals, including several instructive cytokines and/or costimulatory molecules induced by innate immune signaling pathways. Dendritic cells (DCs) are professional antigen-presenting cells in humans. DC subsets respond to pathogens for which they have corresponding TLRs. In humans, TLR7 is expressed on plasmacytoid DCs (pDCs) and in some studies has also been detected on myeloid DCs (mDCs) [12]. The generation of adaptive immunity is dependent upon the maturation of DCs, a process that is mediated in various ways by TLR signaling [12,26,27]. This process begins with the recognition and sequestration of antigen by DCs in peripheral tissue and the subsequent migration to lymph nodes for presentation to naïve T lymphocytes [26] (Figure 2).

Once inside the lymph nodes, DCs seek out naïve antigen-specific T cells and induce their activation and differentiation into effector cells. The first signal in activation of naïve T cells is an antigen-specific signal resulting from the binding of the T cell receptor to the peptide presented by the MHC molecule [26]. The second signal in activation is driven by costimulatory molecules CD80 and CD86 expressed on the DCs, which interact with CD28 on naïve T cells to stimulate T cell proliferation, cytokine production and generation of CTL response [12,28]. This is an example of TLR-mediated control of adaptive response, as hepatic pDC subsets upregulate costimulatory molecules CD40, CD80 and CD86 by TLR4, TLR7 and TLR9 stimulation [29,30]. Specifically, viral ssRNA (a PAMP), is delivered to and is endocytosed by a pDC, and forms the source of the PAMP. Next, engagement of TLR7 on the endosome triggers the signaling pathways resulting in production of specific cytokines and costimulatory molecules that augment adaptive immune responses (Figure 2) [27,31]. Hence, TLR-mediated signaling impacts adaptive immune responses by producing cytokines that mediate T cell differentiation. In the liver, pDCs produce IFN-α, TNF-α, IL-6 and IL-12 in response to ligands for TLR7/9 [29,32]. Consequently, in response to stimulation by a TLR7 agonist, cytokines such as IL-12 drive the induction of T_H_1 responses on T cells [33], which is characterized by the production of IFN-γ by T cells and further activation of cell-mediated immunity, resulting in the activation of phagocytes, antigen-specific cytotoxic T lymphocytes and the release of various cytokines (Figure 2).

Studies using animal models that lack TLR signaling pathways have clearly established the vital role played by TLR7 in antiviral immunity [11,34]. Mice deficient in MyD88, the adaptor protein that mediates signal transduction by TLR7, are not capable of signaling through TLRs. As a result, their APCs are not stimulated by TLR agonists [35]. MyD88-deficient mice demonstrated a lack of T cell activation in response to antigen, suggesting impairment in the antigen-specific priming of T cells [27]. Consequently, these mice failed to produce detectable amounts of IFN-γ in response to antigen stimulation [27], suggesting that in the absence of TLR signaling, immune responses are severely impaired and unable to generate an effective T_H_1-dependent immune response. In addition, MyD88-deficient DCs treated with antigen did not induce production of CD80, CD86, MHC class II or IL-12, which resulted in an inability of DCs to mature and led to inefficient priming of naïve T cells [27]. These results support the theory that innate immune recognition by TLRs is required for induction of accessory signals required for the activation of adaptive immune response and inducing T_H_1 polarization by expressing IL-12 on APCs [27,36], an event critical for the induction of DC maturation, the generation of T_H_ subsets and the activation of the adaptive immune response against viruses.

### Hepatitis C infection

An estimated 4.1 million people are infected with hepatitis C (HCV) in the United States, with up to 180 million people infected worldwide [37,38]. Approximately 60-85% of individuals infected with HCV will eventually develop chronic hepatitis C infection [39-41]. Although chronic infection is usually asymptomatic, it can lead to chronic liver disease, including cirrhosis, hepatocellular carcinoma and ultimately liver failure [37,42]. In the United States, complications from HCV are the leading indication for liver transplantation [43]. Currently, immune-based therapies including interferon still have a role in the eradication of HCV. The standard of care for HCV is rapidly evolving as safer, more effective therapies become available. Currently, the preferred regimen for HCV genotype 1 infection is a 12-week course of sofosbuvir (an HCV NS5B polymerase inhibitor) in combination with pegylated interferon and ribavirin [44], which resulted in a 90% sustained virological response in phase III clinical trials [45]. The treatment paradigms will likely evolve to include interferon-sparing treatment options for most patients over the next few years.

#### Plasmacytoid dendritic cells and TLR signaling in HCV infection

Many viruses have evolved mechanisms to subvert host immune responses and HCV is no exception. The HCV NS3/4A protease inhibits TLR3 and RIG-I mediated signaling in infected hepatocytes [46], thereby blunting type I interferon responses in these cells. The robust induction of interferon stimulated genes (ISGs) in the livers of HCV-infected patients [23] suggests that cells other than infected hepatocytes may drive type I interferon induction. *In vitro* work done in Huh 7.5 cells suggest that pDCs in HCV-infected livers stimulate type I interferon via TLR7 signaling without becoming infected [47]. Thus, cell-cell interaction should be required in order for pDCs to endocytose HCV RNA particles from infected hepatocytes. Recent work suggests that this cell-cell recognition is facilitated by CD81/CD9 tetraspanins [48]. In summary, these data suggest that TLR7 mediated signaling plays an important role in immune responses in HCV-infected patients, and that exogenous TLR7 agonists may have a role in management of chronic HCV infection.

#### TLR agonists for chronic hepatitis C infection

As treatment for HCV is slowly evolving from an immune based interferon-containing therapy to an interferon-free, all oral, directly acting antiviral (DAA) therapy, much more needs to be learned about the correlates of HCV clearance. Induction of hepatic type I interferons seems to be a reliable early indicator of sustained virologic response to DAA-based therapies [49]. In this regard, HCV therapy can be optimized by enhancing an endogenous hepatic interferon response by using TLR agonists. Hence, the evaluation of novel immunomodulatory strategies, such as the use of TLR7 agonists, which result in an induction of endogenous type I interferon response, is warranted in maximizing the effectiveness of HCV therapeutics.

In a proof of concept study isatoribine, a TLR7 agonist, was shown to cause a significant reduction of plasma HCV RNA in previously untreated HCV-infected individuals [50]. Administration of the TLR7 agonist isatoribine was shown to significantly increase viral clearance by inducing an immune antiviral response, as indicated by dose-dependent changes in immunologic biomarkers [50]. Treatment was well tolerated and viral reduction occurred in both genotype 1 and non-genotype 1 HCV infection [50]. Since this initial trial, small-molecule TLR7 agonists have become a focus of clinical trials for the treatment of chronic HCV infection. In a multi-center, phase IIa study, resiquimod, a TLR7/8 agonist was taken orally for 4 weeks resulted in a transient viral reduction, but serious adverse events were reported consistent with IFN-α induction, including fever, headache, shivering and lymphopenia [51]. As a result of the systemic effects of the drug, resiquimod was withdrawn from clinical testing for HCV but was further examined as a topical agent for HPV [52]. Another agent, PF-4878691, effectively induced immune biomarkers and IFN responses in a dose-dependent manner, but its antiviral effect was only produced at doses associated with serious adverse events [53,54]. An oral isatoribine prodrug, ANA773, was administered to HCV-infected patients every other day for 28 days (800 mg, 1200 mg, or 1600 mg) or for 10 days (2000 mg). A significant reduction of serum HCV RNA levels was seen in the 2000 mg-dosed group. The drug was generally well tolerated and most of the reported adverse events symptoms disappeared rapidly during continued dosing, resulting in no dose reductions or discontinuations. Thus, ANA773 is the first oral TLR7 agonist to induce a dose-related antiviral response leading to a significant decrease in serum HCV RNA levels without the concurrent induction of prohibitive systemic side effects [55]. Hence, it is plausible that viral eradication may be enhanced using a strategy that optimally targets the host innate immune system by using an oral selective TLR7 agonist to induce a direct antiviral effect (type I interferon signature) and an indirect effect (enhancing HCV-specific immunity by cross-linking innate and adaptive immune system), resulting in rapid eradication of HCV without a myriad of adverse events.

### Hepatitis B infection

Hepatitis B (HBV) is an enveloped, double stranded DNA virus that can be transmitted perinatally, percutaneously, or through close person-to-person contact [56]. An estimated 350 million individuals are chronically infected with hepatitis B worldwide [57], with approximately 1.25 million HBV carriers living in the United States [56,58]. HBV carriers are at an increased risk of liver damage, including cirrhosis, hepatic decompensation and hepatocellular carcinoma [59,60]. Hepatitis B virus is self-limited in 90-95% of adults. The remainder will develop chronic hepatitis B infection, as demonstrated by the presence of HBsAg+, serum HBV DNA, persistent or intermittent elevation in ALT/AST levels and evidence of necroinflammation.

#### Treatment for HBV

The current goal of chronic hepatitis B treatment is to achieve long lasting suppression of HBV replication to prevent cirrhosis, hepatic failure and HCC. Currently, immune based treatment of chronic HBV infection consists of IFN administration. Nucleoside analogues (NA), mainly tenofovir and entecavir, can achieve long-term suppression of HBV. Complete response to treatment, also known as HBV clearance, consists of an HBsAg seroconversion, normalization of ALT/AST, absence of serum HBV DNA and absence of covalently closed circular DNA (cccDNA). Complete response is rare with the use of NA alone, but can be rarely achieved through the use of immune-based therapy, or via rare spontaneous clearance. In the absence of a cure, long-term suppression of HBV is required to prevent progression of liver disease. However, long-term suppressive therapy with NA is associated with emergence of resistance and toxicities [61]. Some nucleoside analogues have a low barrier to resistance and once antiviral-resistant HBV mutants are selected they are typically retained in the virus population indefinitely [62]. Even in the setting of successful viral suppression and normal ALT/AST levels, it is uncertain what long-term risk of sequelae like HCC is in these patients. Although chronic HBV infection may be cleared without induction of innate immune responses [63] there is proof of principle data available that immune-based treatment such as interferon-α is capable of achieving protective immunity in some treated patients [64]. This offers hope that similar strategies, that are safer, more tolerated and orally available could be vital in accomplishing a “functional cure” of HBV.

#### TLR agonists for chronic hepatitis B infection

Studies have shown that liver of HBV transgenic mice produce IFN-α, −β and -γ to inhibit hepatitis B replication, suggesting that HBV replication can be controlled by activation of innate immune response in the liver [65] When HBV transgenic mice were injected with ligands specific for TLR 2 through 9, HBV replication was nearly eliminated after a single injection of 10 μg of each ligand except for TLR2 ligands. The inhibition of HBV replication was essentially noninflammatory and noncytopathic and was accompanied by the induction of IFN-α and IFN-β [65]. The inhibition of HBV replication was accomplished at a post-transcriptional level by suppressing the assembly or stability of HBV RNA-containing capsids, where HBV DNA synthesis is known to occur [65,66]. These findings provide evidence that TLR activation directly inhibits HBV replication [18,67]. However, HBV may evade innate recognition by TLRs as a strategy to escape the innate immune response by disrupting TLR expression, resulting in inhibition of TLR signaling cascades [18,67]. It has been reported that the expression of TLRs in hepatocytes and other cells is down regulated in the presence various HBV viral products [68-73]. Although HBV circumvents endogenous type I interferon pathways, it is plausible that exogenous interferon induction by using a TLR7 agonist may reinstate interferon-α responses. When combined with a strategy that results in maximal suppression of HBV replication in vivo using NAs, exogenous IFN stimulation via TLR agonists may result in development of protective immunity. Several studies have shown that long-term suppression of HBV using NAs results in partial reconstitution of adaptive immunity. Furthermore, TLR7 mediated signaling may also contribute to adaptive immune responses [12]. In this regard, an adjuvant therapy using a TLR agonist may be able to accelerate this process of immune reconstitution and aid in functional cure or HBsAg seroconversion both by innate and adaptive immune signaling.

There are other potential benefits in the use of TLR agonists for treatment of chronic HBV infection. First, TLR agonists are available as oral compounds enabling rapid uptake by liver. Pre-systemic activation by TLR agonists via gut absorption into portal circulation may allow for optimal, lower doses of adjuvant therapy to minimize adverse events. Second, they may be co-formulated with other NAs as a single pill. Third, TLR7 agonists are selective and do not activate the TLR8 pathway, thus eliminating the negative affects of TNF-α activation. Finally, similar to injected IFN, TLR agonists induce IFN production, triggering the production of cytokines to facilitate intracellular communication and cellular trafficking. However, through the use of TLR agonists this antiviral state can be induced at the local level, pre-systemically rather than systemically, eliminating the adverse events associated with systemic innate immune activation.

Recent studies have shown that TLR7 agonist administration results in an augmentation of downstream signaling of interferon stimulated genes (ISGs) without systemic IFN-α related adverse events. In particular, the induction of ISG15 and CCL8 mRNA have been implicated as markers of pre-systemic immune response in response to TLR7 agonist stimulation [74]. Recently, in chronically infected chimpanzees, GS-9620, a selective oral agonist of TLR7, induced prolonged suppression of HBV DNA in both the serum and liver [75]. GS-9620 administration induced the production of IFN-α and various cytokines and chemokines. In addition, it activated all lymphocyte subsets to induce ISGs [75]. HBV DNA was reduced in addition to serum levels of HBsAg, HBeAg and HBV antigen positive hepatocytes [75]. In early studies of oral GS-9620 in healthy volunteers, oral doses (single dose of 0.3, 1, 2, 3, 4, 6, 8, or 12 mg) were well absorbed and well tolerated in doses up to 12 mg. Adverse events associated with IFN treatment were seen subjects who received 8 mg or 12 mg dose and serum IFN-α was only detected at these doses although activation or ISGs were seen at doses ≥2 mg [76].

## Conclusions

Optimal induction of innate and adaptive immunity contribute to host defenses against viral pathogens such as hepatitis B and C. Endogenous induction of type I IFNs contributes to the clearance of these viruses, hence the appeal of selective TLR7 agonists,which generate endogenous type I interferons and could thus aid in augmenting spontaneous and therapeutic clearance of hepatitis viruses. Adjunctive treatment with such agents may allow for shorter, less toxic, and less expensive courses of antiviral therapy. This approach, if successful, will have a tremendous impact on the public health burden of chronic liver diseases, wherein HCC and death due HCV and HBV infections are common.

## Abbreviations

TLR: Toll-like receptors; PRR: Pattern recognition receptor; HBV: Hepatitis B virus; HCV: Hepatitis C virus; PAMP: Pathogen associated molecular patterns; IL: Interleukin; TIR: Toll/interleukin-1 receptor; MyD88: Myeloid differentiation primary-response protein; IRAKs: IL-1R associated kinases; TNFR: Tumor necrosis factor receptor; TRAF6: TNFR-associated factor 6; TGF-B: Transforming growth factor B; TAK1: TGF-B activated kinase; NF-κB: Nuclear factor kappa B; IFR7: IFN regulatory factor 7; TNF-α: Tumor necrosis factor alpha; MHC: Major histocompatibility complex; APC: Antigen presenting cell; pDC: Plasmacytoid dendritic cell; mDC: Myeloid dendritic cell; IFN: Interferon; TH1: Type 1 T helper cells; SVR: Sustained virologic response; ISG: Interferon stimulated genes; RIG-1: Retinoic acid inducible gene 1; HBsAg: Hepatitis B s antigen; NA: Nucleoside analogues; HCC: Hepatocellular carcinoma; HBeAg: Hepatitis B e antigen; DAA: directly acting antiviral.

## Competing interests

The authors declare that they have no competing interests.

## Authors’ contributions

EF performed literature search, wrote the primary manuscript, SK and BG assisted with concept and design of the manuscript, proof read the manuscript and RT designed the manuscript concept and wrote the manuscript. All authors read and approved the final manuscript.
